# The significance of low PU.1 expression in patients with acute promyelocytic leukemia

**DOI:** 10.1186/1756-8722-5-22

**Published:** 2012-05-08

**Authors:** Xuehua Zhu, Hui Zhang, Maoxiang Qian, Xujie Zhao, Wentao Yang, Ping Wang, Ji Zhang, Kankan Wang

**Affiliations:** 1 State Key Laboratory of Medical Genomics, Institute of Health Sciences, Shanghai Institutes for Biological Sciences, Chinese Academy of Sciences (CAS) & Shanghai Jiao Tong University School of Medicine (SJTUSM) and Shanghai Institute of Hematology, Rui-Jin Hospital, SJTUSM, Shanghai, 200025, China

**Keywords:** PML-RARα, PU.1, Acute promyelocytic leukemia

## Abstract

**Background:**

Although the importance of the hematopoietic transcription factor PU.1 in acute myeloid leukemia (AML) has been demonstrated, the expression of PU.1 in acute promyelocytic leukemia (APL) patient samples awaits further investigation. The current study used APL patient samples to assess the expression pattern of PU.1 in the initiation and progression of APL.

**Findings:**

We used real-time RT-PCR to compare PU.1 expression between *de novo* APL patient samples and normal blood specimens, and the results indicated that PU.1 expression was significantly lower in newly diagnosed APL patient samples as compared to normal hematopoietic cells. Further evidence showed a significant inverse correlation between the expression level of PML-RARα and that of PU.1. In addition, we analyzed the correlation between PML-RARα and PU.1 expression in a large population of AML patients retrieved from the expression profiles. The results showed that PU.1 expression was lower in patients with APL than other AML subtypes and there was also a trend towards increasing PU.1 expression from AML-M0 to AML-M5, with the exception of AML-M3 (APL). These observations suggested that PU.1 expression was reduced by PML-RARα in APL patients. Furthermore, we measured PU.1 expression in APL-initiating cells isolated from *de novo* APL patients by side population cell analysis and found that suppression of PU.1 expression occurred concurrently with PML-RARα expression, indicating the pivotal role of PU.1 in APL initiation.

**Conclusion:**

Our findings provide evidence that low PU.1 expression in APL patients is required for disease initiation and progression.

## Introduction

Acute promyelocytic leukemia (APL) is typified by the t(15;17) translocation, which generates the PML-RARα fusion protein and produces a beneficial response to all-*trans* retinoic acid (ATRA) and arsenic trioxide [1]. At the molecular level, PML-RARα affects the normal functions of wild-type PML and RARα signaling. However, PML- or RARα-deficient mice display few obvious defects [2,3]. Furthermore, in PML-RARα transgenic mice, on average, only 30% develop APL after a long latent period of observation [4], suggesting that APL development may require additional genetic events that are indispensable for myeloid differentiation.

Recently, we demonstrated that PML-RARα interferes with the function of PU.1, which results in a block of downstream PU.1 signaling [5]. In addition, the study by Mueller BU *et al.* also reported that PU.1 is suppressed by PML-RARα and that ATRA treatment is capable of restoring PU.1 expression [6]. Although these findings have been confirmed using cell lines, there is a growing evidence to suggest that cell lines do not fully recapitulate the biology of human disease. Therefore, to most accurately examine the role of PU.1 in APL, investigation into the expression profile of PU.1 in APL patient samples is required.

## Materials and methods

### Patient samples and human umbilical cord blood

The study was approved by the Ethics Committee of Ruijin Hospital affiliated to Shanghai Jiaotong University School of Medicine and was adherent to the regulations of the declaration of Helsinski. Bone marrow specimens were obtained after receiving informed consent from patients at the time of their diagnosis with *de novo* APL (Table 1). Peripheral blood specimens were obtained from healthy volunteers with informed consent. Umbilical cord blood (UCB) specimens were obtained with informed consent from volunteer donors attending the obstetrics department at Ruijin Hospital.

**Table 1:** Characteristics of the patient population

Patient no	Age	Sex	Blasts (%)	WBC (×10^9^/L)	HGB (g/L)	Platelets (×10^9^/L)	Karyotype
APL1	53	M	92.5	4.9	105	64	46,XY,t(15;17)(q22;q12)[7]/ 46,XY[2]
APL2	47	M	82	3.3	100	7	46,XY,t(15;17)(q22;q12)
APL3	31	M	91	15.1	100	17	ND
APL4	65	M	95.5	ND	ND	ND	46,XY,t(15;17)(q22;q12)[8]/ 46,XY[3]
APL5	34	M	97.5	2.04	57	45	46,XY,t(15;17)(q22;q12)
APL6	51	F	91	3.9	78	71	46,XX,t(15;17)(q22;q12)[9]
APL7	58	F	85.5	5.6	101	62	46,XX,t(15;17)(q22;q12)[10]/ 46,XX[11]
APL8	34	F	98	7.5	117	34	46,XX,t(15;17)(q22;q12)[10]/ 46,XX[11]
APL9	40	M	85.5	5.7	69	30	46,XY,t(15;17)(q22;q12)[12]/ 46,XY[3]
APL10	24	M	91	2.5	122	60	46,XY,t(15;17)(q22;q12)

### Cell preparation

Mononuclear cells and granulocytes were isolated using Ficoll-Paque (Lymphoprep™, Fresenius Kabi Norge AS, Norway) density gradient separation. CD34^+^ cells were isolated from UCB specimens using a high magnetic gradient MiniMACS purification system (Miltenyi, Sunnyvale, CA).

### Side population analysis of fresh APL and UCB specimens

Side population (SP) cell analysis was performed according to the protocol from Goodell's laboratory [7] with minor modifications.

### Real-time RT-PCR

Total RNA was extracted from cells using an RNeasy Kit from Qiagen (Chatsworth, CA). Reverse transcription was performed using the Superscript II reagent set (Invitrogen, Carlsbad, CA) with random hexamer primers. Quantitative real-time PCR was performed using an ABI Prism 7900HT detection system (Applied Biosystems, Foster City, CA). The relative expression level for each target in comparison to the internal control GAPDH was calculated using the following equation: ΔCt = Ct (target) - Ct (GAPDH), where the relative mRNA expression = 2^-ΔCt^ × 100. Each assay was performed in triplicate.

The expression of PML-RARα, PU.1 and GAPDH mRNA in cells was analyzed by real-time PCR with the following primers corresponding to distinct sequences: sense (5’-AAGTGAGGTCTTCCTGCCCAA-3’) and antisense (5’-GGCTGGGCACTATCTCTTCAGA-3’) for PML-RARα; sense (5’-AGAAGAAGATCCGCCTGTACCA-3’) and antisense (5’-GTGCTTGGACGAGAACTGGAA-3’) for PU.1; sense (5’-GAAGGTGAAGGTCGGAGTC-3’) and antisense (5’- GAAGATGGTGATGGGATTTC-3’) for GAPDH.

### Gene expression analysis

The raw gene expression data and clinical data from four cohorts of AML patients were provided by other research groups [8-11]. To perform interarray comparisons, the CEL files were analyzed using Affymetrix MAS 5.0 software. The PU.1 expression level relative to that of GAPDH was calculated as log_2_^(10000 × expression level of PU.1 / expression level of GAPDH)^. Two-tailed *t* tests were used to validate the significance of the observed differences.

## Results

### PU.1 expression is significantly lower in APL patient samples in comparison to normal hematopoietic cells

**Figure 1:**
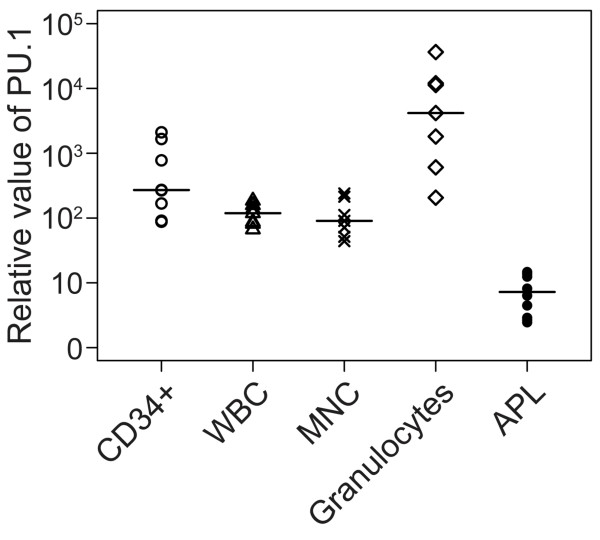
**Lower level of PU.1 mRNA expression in*de novo*APL patients in comparison to normal hematopoietic cells.** The relative PU.1 expression, as compared to that of the reference gene GAPDH, in leukemic cells freshly isolated from APL samples (Table 1), normal peripheral blood cells (WBCs, MNCs, and granulocytes) and CD34^+^ cells from human UCB specimens was determined using real-time RT-PCR. Each symbol represents the average value from an individual patient or healthy subject, and the lines indicate the median value.

We first examined PU.1 mRNA expression in mononuclear cells from 10 newly diagnosed APL patients with high percentages of blasts (> 80%, mean 91%) (Table 1). The peripheral blood cells isolated from 7 healthy volunteers and the normal hematopoietic stem cell-enriched CD34^+^ cells isolated from 8 fresh human UCB specimens served as controls. As shown in Figure 1, PU.1 expression was significantly lower in the primary APL samples in comparison to normal hematopoietic cells, including white blood cells (WBCs), mononuclear cells (MNCs), granulocytes and immature progenitor cells (*p* = 7.6 × 10^-7^, 4.6 × 10^-4^, 1.5 × 10^-7^ and 0.015, respectively).

### PU.1 is expressed at lower levels in patients with APL as compared to other AML subtypes

**Figure 2:**
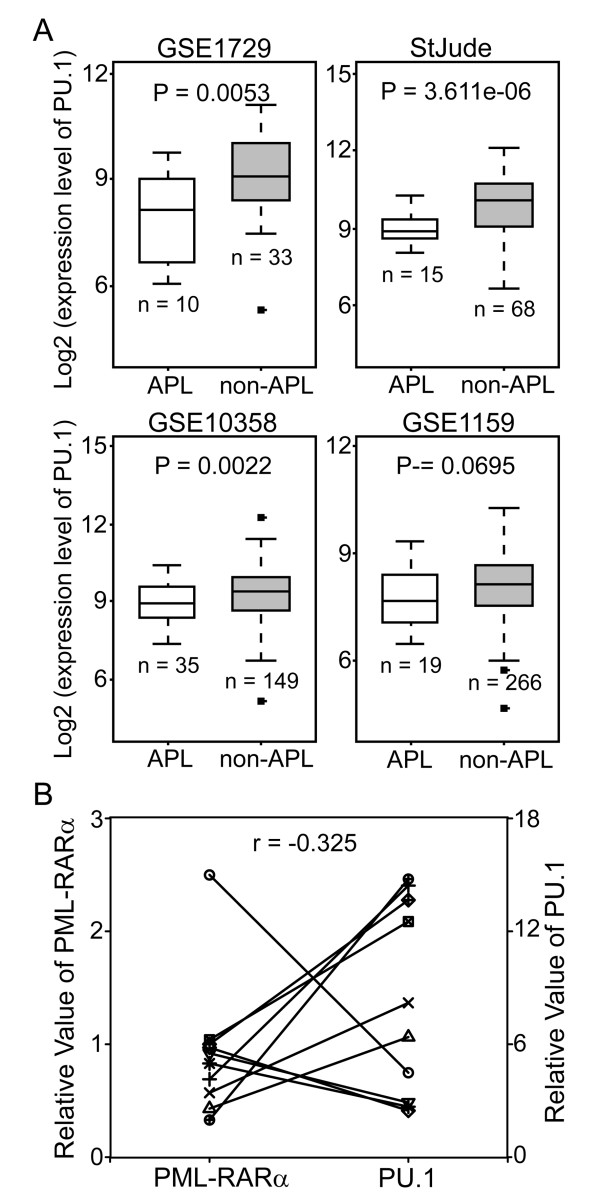
**Lower levels of PU.1 expression in APL patients as compared to non-APL AML patients. (A)** Four representative gene expression profiling data sets were retrieved, including GSE1729, StJude, GSE10358 and GSE1159. White boxes indicate the expression levels of PU.1 in APL patients, and gray boxes represent the levels of PU.1 in non-APL (other AML subtypes) patients. The differences between the two groups were assessed using the two-tailed *t* test. The *p*-values are shown in the panels. **(B)** The inverse correlation between PML-RARα and PU.1 mRNA expression was assessed by quantitative real-time RT-PCR using 10 fresh APL patient samples (Table 1). The correlation coefficient for PML-RARα versus PU.1 was r = −0.325. Each symbol represents the average values from individual patients.

To draw more solid conclusions, we retrieved four datasets containing the expression profiles of 595 AML patients, 79 APL patients and 516 patients with other AML subtypes [8-11] and compared PU.1 expression between APL patients and non-APL AML patients. A genome-wide gene expression analysis of blasts from AML patients revealed that PU.1 was clearly expressed at a lower level in APL patients as compared to non-APL patients (Figure 2A), indicating that PU.1 expression might be specifically reduced by PML-RARα in APL. To further examine the relationship between PML-RARα and PU.1, we compared the levels of PML-RARα and PU.1 mRNA in the group of *de novo* APL patients shown in Table 1. As expected, although there was considerable variation from patient to patient, a significant inverse correlation (r = −0.325) was observed between the expression level of PML-RARα and PU.1 (Figure 2B), which is consistent with the findings by Mueller BU *et al.*[6]. These results indicate that PML-RARα suppresses PU.1 expression in APL patients.

### PU.1 expression is particularly repressed in APL

**Figure 3:**
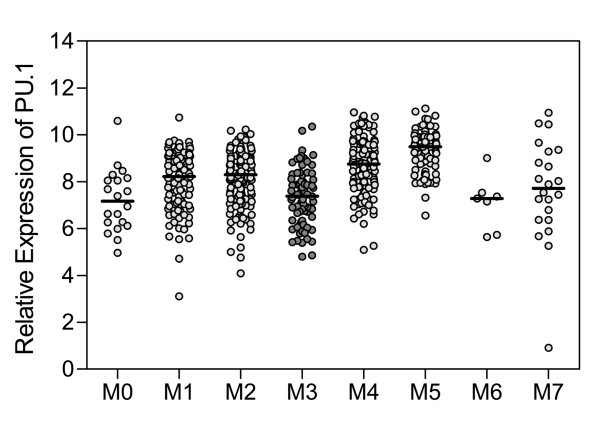
**Particularly low levels of PU.1 expression in patients with APL as compared to other AML subtypes**. AML patients were divided into eight subtypes (M0, M1, M2, M3, M4, M5, M6 and M7) according to FAB classification. The expression levels of PU.1 in the eight subtypes were evaluated. The four profiling data sets of AML patients, including GSE1729, StJude, GSE10358 and GSE1159, were combined. The PU.1 level relative to that of GAPDH was calculated as described in the Materials and methods.

Furthermore, we evaluated PU.1 expression for each subtype of AML according to the French-American-British (FAB) classification. As shown in Figure 3, low PU.1 expression was observed in the M3 subtype (APL). Interestingly, in addition to the M3 subtype, low PU.1 expression was also observed in the M0, M6 and M7 subtypes. As a low level of PU.1 is a prerequisite for the differentiation of common myeloid progenitors (CMPs) to megakaryocyte/erythroid progenitors (MEPs) [12], it is reasonable that PU.1 levels would be lower in the M6 (acute erythroleukemia) and M7 (acute megakaryocytic leukemia) subtypes. The observation that PU.1 expression appeared low in the M0 subtype (the most immature FAB subtype) may be explained by the fact that PU.1 expression is low in bone marrow cells lacking definitive signs of myeloid differentiation [12]. In addition, Figure 3 shows a trend towards increasing PU.1 expression from M0 to M5 with the exception of M3, which supports the notion that PU.1 expression is specifically repressed by PML-RARα.

### PU.1 is suppressed in APL initiation

**Figure 4:**
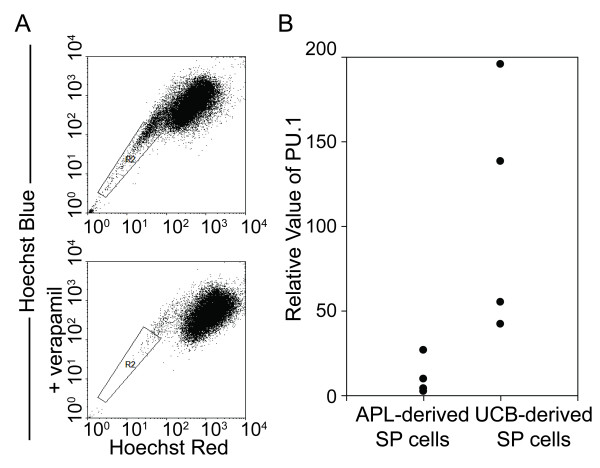
**Lower levels of PU.1 expression in APL-initiating cells.(A)** SP cell analysis. The SP cells were gated in the R2 box and were detected following Hoechst 33342 and verapamil co-treatment. Hoechst staining of APL mononuclear cells in the absence or presence of verapamil is shown in the upper and lower panels, respectively. The FACS profiles represent the results of the flow cytometry analysis of SP cells from APL samples. **(B)** Lower PU.1 levels in APL-derived SP cells as compared to UCB-derived SP cells. The PU.1 mRNA expression levels of SP cells from UCB specimens or primary APL samples were measured using real-time RT-PCR and were normalized to GAPDH expression. Each symbol represents the average value from an individual patient or UCB specimen.

Growing evidence suggests that leukemia-initiating cells are responsible for initiating and sustaining the growth of the disease. Therefore, we evaluated the expression of PU.1 in APL-initiating cells. Unlike other subtypes of AML, most APL cells lack the CD34^+^ surface marker [13]. Zheng X *et al.* recently isolated SP cells from APL cells [14], and these cells have been reported to be highly enriched for cancer-initiating cells [15]. Therefore, we isolated SP cells from 4 *de novo* APL patient samples (Figure 4A), and SP cells from 4 human UCB specimens were included as controls. As shown in Figure 4B, the expression level of PU.1 was markedly lower in SP cells from primary APL samples in comparison to those from UCB specimens, indicating that the expression of PU.1 during APL initiation is reduced by PML-RARα.

## Discussion

PU.1 not only plays an importance role in normal hematopoiesis but is also strongly implicated in leukemogenesis. By comparing PU.1 expression between APL patients and normal blood samples, we found that PU.1 expression was significantly lower in APL patients. By analyzing the expression pattern of PU.1 across a large scale of AML patients, we found that PU.1 expression was significantly lower in patients with APL compared to other AML subtypes. These observations may have been the result of the distinctive expression of PML-RARα, which suggests a connection between PML-RARα expression and the decreased expression of PU.1 in APL. Taken together, these data indicate that low PU.1 expression may be a contributing event in APL.

The level of PU.1 expression is critical for hematopoietic lineage commitment and maturation [12]. PU.1 expression is low in hematopoietic stem cells (HSCs), and is upregulated at the CMP stage. CMPs with relatively high levels of PU.1 are mainly committed to the granulocyte and macrophage lineages, whereas those with relatively low levels of PU.1 are committed to the erythrocyte and megakaryocyte lineages [16]. AML is characterized by the blockage of myeloid differentiation at different stages, and FAB classification (M0 to M7) is generally performed according to the type of cells from which leukemia developed as well as their degree of maturity. The observed trend towards increasing PU.1 expression from M0 to M5 (except for M3) may reflect differentiation-related differences in PU.1 expression. Lower PU.1 expression was detected in undifferentiated (M0) AML confirms the finding that low PU.1 expression is present in blasts with minimal differentiation [12]. The intermediate PU.1 expression observed in myeloid (M1/2) AML and the high PU.1 expression observed in myelomonocytic (M4/5) AML are likely associated with the stage at which myeloid differentiation is blocked. Moreover, in regards to the increasing expression from M0 to M5, PU.1 expression in APL (M3) was exceptional and was detected at a significantly lower level, which was likely attributable to distinctive PML-RARα expression at this stage. *In vivo* animal studies have also shown that functional disruption of PU.1 or a graded reduction in its expression blocks myelomonocytic differentiation or maturation, resulting in the accumulation of myeloid blasts and, thus, the genesis of myelogenous leukemia [17,18]. The differentiation of preleukemic cells in these mice was mostly blocked at the immature granulocytic stage, which suggests that other abnormalities may also influence PU.1 expression. Indeed, AML1-ETO has been reported to suppress PU.1 expression [19]. Although PU.1 expression was greater in the M2 stage than the M3 stage, this may have been due to the fact that 70–80% of M2 do not have AML1-ETO expression [20]. A more detailed classification for distinguishing between cell types would help to confirm the role of PU.1 in the development of AML. In addition, low PU.1 expression in erythrocytic (M6) and megakaryocytic (M7) AML supported the observation that PU.1 expression was reduced in the erythrocyte and megakaryocyte lineages [12]. Taken together, our data demonstrate that PML-RARα expression together with reduced PU.1 expression is a characteristic of APL.

Numerous studies have demonstrated that the initiation of APL requires the expression of PML-RARα [21]. However, PML-RARα alone is not sufficient to induce APL [22-24]. Walter *et al.* demonstrated that transgenic mice expressing PML-RARα frequently develop APL in association with the deletion of PU.1 [25]. Moreover, the penetrance rate for APL development was significantly increased when PML-RARα mice were crossed with PU.1+/− mice [25]. Consistent with the above observations, our study found that PU.1 expression was lower during APL initiation using SP cells isolated from APL patient samples.

In conclusion, our data reveal the expression pattern of PU.1 in APL patient samples and provide additional clues about the mechanisms in the initiation and progression of APL. Therefore, we conclude that the formation of PML-RARα and the subsequent suppression of PU.1 expression are critical for the initiation and progression of APL.

## Abbreviations

APL = Acute promyelocytic leukemia; AML = Acute myeloid leukemia; ATRA = All-trans retinoic acid; UCB = Umbilical cord blood; SP = Side population; WBC = White blood cell; MNC = Mononuclear cells; CMP = Common myeloid progenitor; FAB = French-American-British; MEP = Megakaryocyte/erythroid progenitor; HSC = Hematopoietic stem cell.

## Competing interests

The authors declare that they have no competing interest.

## Authors' contributions

XHZ designed the study, performed experiments and wrote the manuscript; HZ and XJZ recruited samples and performed experiments; WTY and MXQ participated in the statistical analyses; PW performed experiments; JZ and KKW designed the study, interpreted the results and wrote the manuscript. All authors read and approved the final manuscript.
